# Characterisation of IgA Nephropathy in an Australian Cohort

**DOI:** 10.1155/ijne/9976879

**Published:** 2025-03-27

**Authors:** Shriram Swaminathan, Nithya Neelakantan, Henry Bryant, Pimvara Rattanamastip, Gagandeep Sandhu, Bobby Chacko

**Affiliations:** ^1^Nephrology and Transplantation Unit, John Hunter Hospital, Newcastle, New South Wales, Australia; ^2^Fay W. Boozman College of Public Health, University of Arkansas for Medical Sciences, Little Rock, Arkansas, USA; ^3^Anatomical Pathology, John Hunter Hospital, Newcastle, New South Wales, Australia; ^4^Nephrology and Transplantation Unit, John Hunter Hospital, School of Medicine and Public Health, University of Newcastle, Newcastle, New South Wales, Australia

**Keywords:** IgA nephropathy, kidney failure, outcomes, predictor, prognosis

## Abstract

**Aim:** This retrospective cohort study aims to evaluate the prognostic factors for progression of immunoglobulin A nephropathy (IgAN) to kidney failure (defined as the initiation of kidney replacement therapy or death) and all-cause mortality in an Australian population.

**Methods:** We conducted a retrospective analysis of 363 individual patients with biopsy-proven IgAN over a 21-year period (2000–2020) in the Hunter Region of New South Wales. Demographic data, comorbidities, biopsy features and biochemical markers were collected for a minimum of 12 months following biopsy diagnosis. A multivariable analysis using Cox regression was performed to examine their association with renal progression.

**Results:** A total of 104 patients met the inclusion criteria and were followed for a median of 72 months. The cohort had a mean age at presentation of 45 years, with a predominantly male population. Most patients presented with haematuria and non-nephrotic range proteinuria. We stratified patients into three risk categories: low risk, intermediate risk, and high risk. Twenty-eight patients (26.92%) developed kidney failure and 15 patients (14.4%) experienced a > 20 mL/min eGFR decline within the first 12 months. The multivariable analysis revealed the following key factors associated with kidney failure: additional renal pathology on biopsy (HR 3.90, 95% CI 1.63–9.29), proteinuria (HR 1.15, 95% CI 1.02–1.29) and moderate-severe interstitial fibrosis/tubular atrophy (T2) (HR 7.00, 95% CI 2.32–21.05). There were 17 deaths (16.3%) in the cohort, with a mean survival time of 167.8 months (95% CI 152.6–183.1).

**Conclusion:** In contrast to earlier reports from Australia, our findings emphasise that the progression to kidney failure is not uncommon in IgAN. We identified several predictors of the renal progression that are consistent with the previous studies. This highlights the need for a change in clinical management, as IgAN should no longer be considered a benign condition.

**Summary**

• This retrospective cohort study of 104 patients with biopsy-proven immunoglobulin A nephropathy (IgAN) in the Hunter Region of New South Wales, spanning a 21-year period, aimed to identify predictors of kidney failure and disease progression.
• With a median follow-up of 72 months, the study found that 26.92% of patients developed kidney failure and 14.4% experienced a significant decline in eGFR within the first year.
• These findings suggest that the IgAN progression to kidney failure is common and should prompt a shift in clinical management, as the disease is not as benign as previously believed.

## 1. Introduction

IgA nephropathy (IgAN) is the most common form of primary glomerulonephritis in the world affecting approximately 200,000–350,000 patients annually [1]. It is reported that 30% of patients with IgAN over the age of 30 will progress to kidney failure within 20 years of the initial renal biopsy [1]. However, local Australian data on risk factors for disease progression and overall mortality remain limited and require further investigation.

In this study, we analysed the histopathologic data and subsequent clinical and biochemical progression of 104 patients at our local unit over a retrospective 21-year period. Our goal was to evaluate the prognostic factors for the progression to kidney failure, mortality risk factors and renal survival rates.

## 2. Methods

This study was a retrospective cohort study of patients with biopsy-proven IgAN in the Hunter Region of New South Wales. Our inclusion criteria were patients with an initial kidney biopsy confirming IgAN within the study period, who also had a minimum of 12 months of follow-up data for outcome analysis. The end of follow-up was defined as 31st December, 2021, the date of death, or the last available follow-up information for those who lost to follow-up after at least 12 months. The primary outcome for renal survival was defined as the progression to kidney failure. Kidney failure was defined as a composite endpoint, including either (1) progression to Chronic Kidney Disease Stage 5D requiring kidney replacement therapy (dialysis or transplantation) or (2) death attributable to kidney failure without initiation of kidney replacement therapy. Kidney transplantation was recorded but did not influence the determination of the renal survival endpoint, as the initial kidney failure event was the focus of the analysis.

Patients with a diagnosis of IgA vasculitis or Henoch–Schönlein purpura, secondary causes of IgAN, repeat biopsies outside the study period, previous transplant biopsies of native IgA or incomplete data were excluded from the analysis.

We reviewed 1585 renal biopsy records from our nephrology registry, which included 363 individual patients with the diagnosed IgAN between 2000 and 2020. Of these, 259 patients were excluded due to incomplete data regarding demographic factors, comorbidities, medications, biopsy findings, biochemistry, proteinuria or insufficient follow-up for the analysis.

The patients were stratified into three risk groups:

• Group A (low risk): Isolated asymptomatic microscopic haematuria and/or baseline proteinuria < 0.5 g/day
• Group B (intermediate risk): Baseline proteinuria ≥ 0.5 g/day and/or GFR < 60 and/or hypertension > 140/90 mmHg
• Group C (high risk): Acute or rapid loss of GFR and/or crescentic/rapidly progressive glomerulonephritis on biopsy

**Figure 1:**
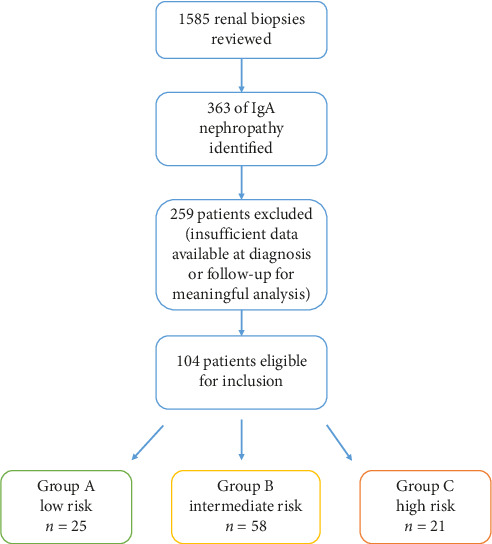
Schematic flowchart of enrolment and stratification of patients.

This risk stratification system was designed to reflect established clinical criteria [2] for IgAN management, guiding treatment decisions between supportive therapy and immunosuppression. A secondary aim was to assess if this traditional risk stratification accurately predicted the progression to kidney failure.  Figure 1 outlines this process as a schematic flowchart.

### 2.1. Statistical Analysis

Demographic data, comorbidities, biopsy features (including MEST scoring [3]) and biochemical markers such as proteinuria and eGFR were then recorded for a minimum follow-up of 12 months after biopsy. Biopsy results prior to the routine use of MEST scoring were retrospectively assigned a MEST score by a clinical histopathologist.

Data were summarised using descriptive statistics, including number (%) for categorical data, mean (±standard deviation) for normally distributed continuous data or median (25th and 75th percentile) otherwise. The comparisons between progression versus nonprogression to kidney failure for continuous variables were done using independent *t*-test and Mann–Whitney test for normally and non-normally distributed data, respectively. The Pearson Chi-square test or Fisher's exact test was used to compare categorical variables. Renal survival was calculated using the Kaplan–Meier method and compared between groups using the Log-rank test. The relationship between patient variables and progression to kidney failure was examined by Cox regression. The multivariable Cox regression was carried out with factors whose univariate Cox regression test had a *p* value of < 0.05. Although statistically significant, the risk group was not included in the multivariable Cox analysis because it was a derived variable based on proteinuria and other clinical variables. Furthermore, there were no events in the low-risk group, and therefore, we observed convergence issues and extreme CIs for the high-risk group in comparison with the intermediate group. Alternatively, we included baseline proteinuria which was found to be statistically significant in the univariate Cox regression analysis. All analyses were carried out using Stata software Version 16.0, with two-sided test at 5% level of significance.

This study was conducted with approval from the local health district ethics committee, ensuring compliance with ethical guidelines. All patient data were deidentified, stored securely and handled in accordance with institutional policies for privacy and data integrity. Upon study completion, all data were destroyed securely following institutional guidelines.

## 3. Results

### 3.1. Patient Demographics and Renal Survival

Table 1 summarises the demographic data for our cohort of 104 patients who met the study's eligibility criteria. The mean age at presentation was 45 (±17) years with a median age of 43. The cohort was predominantly male (70.2%). Most patients presented with haematuria (83.7%) and non-nephrotic range proteinuria. At presentation, the mean serum creatinine was 161.9 ± 139.5  μmol/L with mean baseline proteinuria of 2.5 g/day (±2.7).

**Figure 2:**
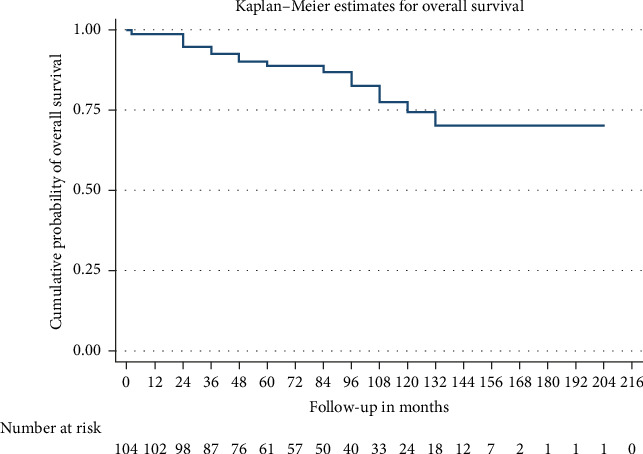
Kaplan–Meier analysis for overall survival. Footnote: Censored by loss to follow-up or the end of the study period.

**Figure 3:**
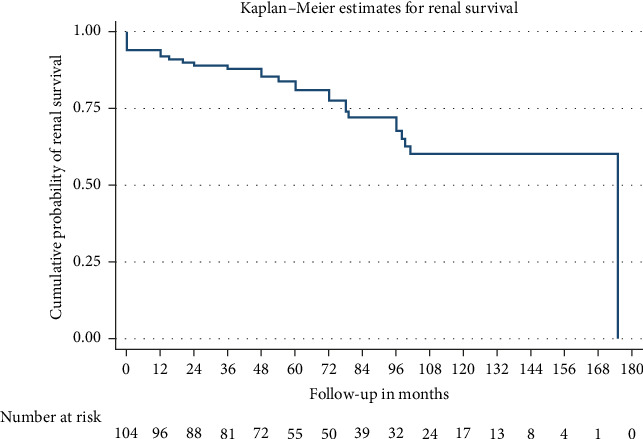
Kaplan–Meier analysis for renal survival. Footnote: Censored by death, loss to follow-up or the end of the study period. Kidney transplantation was recorded for informational purposes but did not affect the determination of the initial kidney failure event or censoring status.

Patients were followed up for a median of 72 months. Among the cohort, 28 (26.92%) developed kidney failure with a mean renal survival time of 129 months (95% CI 114.5–143.4). Fifteen patients (14.4%) experienced a > 20 mL/min decline in eGFR within the first 12 months. Nine of the 28 patients who progressed to kidney failure received a kidney transplant. Despite a decrease in proteinuria levels over time—mean proteinuria was 1.2 g/day (±2.1) at 12 months and 1.5 g/day (±3.8) at final follow-up—renal progression occurred. Seventeen patients died with a mean survival time of 167.8 months (95% CI 152.56–183.1). Figures 2 and 3 present the Kaplan–Meier analysis for overall survival and renal survival. All patients received treatment with renin–angiotensin–aldosterone system (RAAS) inhibitors. Immunosuppressive therapy was administered to 20.2% of patients, with regimens varying over 21 years of the cohort analysis, typically involving systemic corticosteroids and cyclophosphamide.

**Figure 4:**
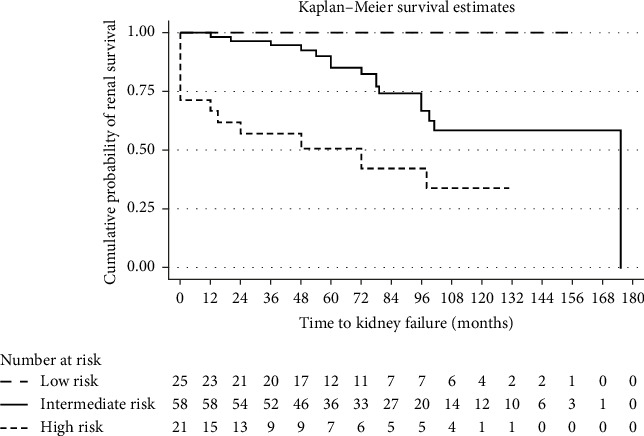
Kaplan–Meier analysis for renal survival stratified across risk group status. Footnote: Censored by death, loss to follow-up or the end of the study period. Kidney transplantation was recorded for informational purposes but did not affect the determination of the initial kidney failure event or censoring status.

When stratified by the baseline risk group, no patients in Group A (low risk) progressed to kidney failure, compared to 16 out of 58 patients (27.6%) in Group B (intermediate risk) and 12 out of 21 patients (57.1%) in Group C (high risk). These differences were statistically significant (*p*=0.001).  Figure 4 shows the Kaplan–Meier analysis of renal survival between these three groups.

### 3.2. Univariate Analysis

Table 2 outlines the clinical, biochemical and histopathologic variables associated with the risk of progression to kidney failure. A statistically significant difference (*p* < 0.05) was observed between the cohort that progressed to kidney failure and those who did not, in relation to several factors. These included risk group status (A, B and C), mesangial cellularity (M), interstitial fibrosis/tubular atrophy (T), baseline proteinuria levels and the presence of additional pathologies on renal biopsy. Additional pathologies identified in biopsies typically included secondary focal segmental glomerulosclerosis and concurrent diabetic or hypertensive changes.

### 3.3. Multivariable Model

Initial multivariate modelling was based on potential factors that were found to be statistically significant up to 5% in the univariate analyses. The modelling process aimed to identify statistically significant predictors, leading to the final inclusion of mesangial cellularity (M), interstitial fibrosis/tubular atrophy (T), presence of additional renal pathology on biopsy and baseline proteinuria. Although the risk group status was significant, it was not included in the multivariable analysis as it was a derived variable based on baseline proteinuria and other clinical parameters.

Risk factors for the progression to kidney failure by the multivariate (Cox regression) analysis are summarised in Table 3. Notable results include T2 (HR 7.00, 95% CI 2.32–21.05), baseline proteinuria (HR 1.15, 95% CI 1.02–1.29) and the presence of an additional renal pathology on biopsy (HR 3.90, 95% CI 1.63–9.29) which were all statistically significant.

## 4. Discussion

In our retrospective cohort study of 104 patients, we demonstrated that 26.9% of patients progressed to kidney failure over a median follow-up of 72 months. The mean age of 45 years and a male predominance in our cohort are consistent with the findings from similar population-based studies on long-term renal prognosis and risk prediction [4, 5].

We stratified patients into low risk, medium risk and high risk based on baseline proteinuria, baseline eGFR and the presence of crescents on biopsy. This traditional stratification approach has been used to guide treatment decisions between supportive therapy and immunosuppression [2]. It allowed for a direct comparison between the groups and facilitated statistical analysis.

Our study found a 5-year renal survival rate of approximately 83.7% and a 10-year renal survival rate of around 74.0% in an Australian population. Contrary to the previous reports, kidney failure is not uncommon in this cohort. For comparison, in 2003, Geddes et al. [6] reported a 10-year renal prognosis of 87% in a similarly sized Australian cohort. However, their UK and Canadian populations had much poorer 10-year renal prognoses of 63.9% and 61.6%, respectively. In earlier Australian studies, such as those by Nicholls et al. [7] in 1984, 5-year and 10-year renal survival rates were 91.5% and 87.5%, respectively. This cohort, although having a similar male predominance, was younger with a mean age of 31.9 years and predominantly presented with haematuria. The authors inferred from their findings that persistent haematuria was linked to a worse prognosis, particularly when associated with crescents on biopsy. In our study, the univariate analysis did not show a significant difference in the progression to kidney failure based on baseline haematuria compared to other risk factors. However, there was insufficient data on ongoing haematuria, as it was not routinely measured during follow-up reviews, which may have impacted our findings.

Similarly, in 1997, Ibels et al. [8] analysed a predominantly male cohort of 174 patients, who mostly presented with persistent microscopic haematuria and subnephrotic range proteinuria, with a mean age of 39 years. They reported a 5-year and 10-year renal survival rate of 92% and 88%, respectively, from the time of biopsy. The main factors associated with the progression in their study included nephrotic range proteinuria and severe renal impairment at presentation. These findings are consistent with our study's observations, where baseline proteinuria and renal impairment were significant risk factors for the progression to kidney failure.

In comparison, studies from non-Caucasian populations, such as Chacko et al.'s study [9] in India, reported a notably poor 10-year renal survival rate of 33%, suggesting a more malignant nature of the disease in that country. These studies, which evaluated patients who initially presented between 1980 and 1990 and were monitored into the early 2000s, found that macroscopic haematuria was a protective factor for the progression.

More recent studies, such as Moryama et al.'s study [10] in Japan, reported a 10-year renal prognosis of 83.6%. Across all these studies, major risk factors for kidney failure included hypertension, lower eGFR at presentation, proteinuria and smoking. Chacko et al. [9] also noted that a significant proportion of patients (55%) presented with nephrotic syndrome. While additional studies have reported uncontrolled hypertension as a risk for kidney failure [11–14], our study did not find a statistically significant association between hypertension and progression to kidney failure. A summary of the above data, comparing similar studies in Australia and the Asia–Pacific region, is shown in Table 4.

In 2023, Pitcher et al. [16] published a retrospective cohort analysis of the UK national registry of rare kidney diseases. Despite having a significantly larger cohort, their study had a very similar median follow-up period of 5.9 years. They found that 50% of patients reached kidney failure or died during the study, with a median kidney survival of 11.4 years compared to 14.6 years in our cohort. Notably, while higher time-averaged proteinuria was linked to worse kidney survival, even patients traditionally considered low risk (proteinuria < 100 mg/mmol) experienced significant rates of kidney failure within 10 years. Overall, our study reports poorer 10-year renal survival outcomes compared to earlier Australian studies. Therefore, in conjunction with the findings from Pitcher et al., it is clear that IgAN remains a disease with poor long-term outcomes. These results challenge previous perceptions of risk factors, highlighting that conditions traditionally considered low risk now show significant potential for the progression. This underscores the importance of the ongoing research and a deeper understanding of the disease.

In understanding IgAN in comparison to other kidney pathologies and risk of kidney failure in an Australian population, we reviewed data from the Australian and New Zealand Dialysis and Transplant (ANZDATA) registry. According to the 44th annual report, diabetic kidney disease remains the most common cause of new patients presenting with kidney failure at 38%, compared to 5% for IgAN [17]. However, IgAN remains the most common cause of primary glomerulonephritis leading to kidney failure in this population, accounting for 30% of primary glomerulonephritis cases causing kidney failure in Australia in 2020 [17].

In terms of overall prevalence, diabetes remains the leading cause of primary renal disease, representing 38% of all dialysis patients, while primary glomerulonephritis is the second most common cause, accounting for 21% of cases [17]. While specific data on IgAN's prevalence among dialysis patients are unavailable, IgAN has consistently been the most common cause of primary glomerulonephritis leading to kidney failure over the past 5 years, accounting for 24%–30% of all GNs [17–21].

Interestingly, a retrospective study of the ANZDATA registry showed that IgAN-associated kidney failure was associated with better dialysis and renal transplant outcomes compared to other causes of kidney failure. The study reported a favourable dialysis patient survival hazard ratio (HR) of 0.63 (95% CI 0.57–0.69) and renal transplant patient survival HR of 0.58 (95% CI 0.45–0.74) when compared to kidney failure controls [22].

In the present study, interstitial fibrosis, baseline proteinuria and the presence of an additional renal pathology on biopsy were statistically significant in being associated with the progression to kidney failure. These results align with the previous models for the IgAN progression including the recently proposed international IgAN risk prediction tool by Barbour et al. [4] Notably, this prediction tool did not incorporate crescents in its modelling due to a lack of statistical significance, and our population also did not show a statistically or clinically significant difference in the presence and severity of crescents and progression to kidney failure.

We note that while high-risk group patients had a more significant rate of the progression to kidney failure at 57.1% compared to the other risk groups, the univariate analysis of the cohort on immunosuppression showed only a 33.3% progression to kidney failure, compared to the overall population progression rate of 26.9%. The decision to use immunosuppression was at the discretion of the individual clinician and was generally based on the presence of crescents on biopsy, the degree of chronicity and, in certain cases, significant proteinuria. A large proportion of the high-risk group had significant chronic changes, where the expected benefit from immunosuppression was minimal.

The role of immunosuppression in the treatment of IgAN remains an ongoing contentious issue. Studies such as the STOP-IgAN trial emphasise the need for robust supportive therapy, including RAAS inhibition, blood pressure control and smoking cessation among other measures [15]. Our study neither suggests that the use of immunosuppression significantly impacts kidney failure outcomes compared to the general cohort nor was this variable found to be a statistically significant predictor of the progression. However, since only 21 patients were treated with immunosuppression, it is difficult to draw definitive conclusions on its benefits. This finding is consistent with another recently published retrospective study by Imai et al., which concluded that long-term low-dose oral steroid treatment may not significantly influence renal prognosis [23].

In our study, the main histopathologic indicators of involvement that showed an independent influence on the progression to kidney failure were mesangial cellularity (M) and interstitial fibrosis/tubular atrophy (T) in the unadjusted analysis. However, T maintained statistical significance on the multivariate analysis. Specifically, a classification of T2 showed the greatest risk for the progression, with a HR of 7.00. This is consistent with the findings of Alamartine et al. [24] who also identified T as having the strongest influence on renal survival.

Our study has several limitations. The primary limitation is its retrospective cohort design, which is constrained by the availability of data for the analysis. Ultimately, only 104 of the 363 potential patients had sufficient data for an adequate analysis, which limits the generalisability of our findings to the broader population. The small sample size reduces statistical power, leading to wide confidence intervals and the potential underestimation of smaller effect sizes. Additionally, biopsy samples obtained before the routine use of the MEST scoring system were retrospectively scored by a clinical histopathologist, which may introduce variability compared to prospective assessments.

A significant concern is the potential for selection bias due to the high proportion of excluded patients (259 out of 363). It is possible that patients with fewer risk factors and better outcomes were less likely to have comprehensive follow-up data, as they may have required fewer clinical visits or investigations over time. Conversely, patients with more severe disease or higher risk factors may have been more likely to have detailed and frequent follow-up, leading to their inclusion in the study. If this is the case, the remaining cohort may overrepresent patients with worse outcomes, potentially skewing the results towards a more pessimistic view of the disease progression. This could limit the external validity of our findings, as the study population may not accurately reflect the broader spectrum of IgAN patients, including those with milder diseases.

While the prospective analysis in an independent population would be ideal, there are practical limitations on the time required to conduct such a study, particularly given the often slow progression of IgAN towards kidney failure. In this study, patients were included based on their diagnosis at the time of biopsy, and the progression was analysed from the point of diagnosis onwards. It is likely that some patients were diagnosed later in the disease process, which may have influenced their measured progression from diagnosis. Additionally, the study population spanned a period before sodium-glucose Cotransporter 2 (SGLT2) inhibitors became widely available in the Australian cohort. Future studies should investigate the impact of SGLT2 inhibitors on the IgAN progression.

Despite these limitations, the study provides valuable insights into predictors of progression in IgAN and highlights the need for larger, prospective studies to validate and expand on these findings. Future research should aim to include a more representative sample of patients, including those with milder disease and better outcomes, to better understand the full spectrum of the IgAN progression and to minimise the risk of selection and information bias.

## 5. Conclusion

In summary, the present study analysed the risk factors for the progression to kidney failure in IgAN from an Australian perspective. Our results continue to support the current body of evidence that the progression to kidney failure is relatively common in IgAN, albeit occurring slowly over a period of years. The study highlights several clinical and histopathologic factors, including interstitial fibrosis/tubular atrophy (T), additional renal pathology on biopsy and baseline proteinuria all of which show significant association with the progression to kidney failure. While these findings contribute new insights into our understanding of IgAN, they are ultimately consistent with the previous evidence.

## Figures and Tables

**Table 1:** Patient demographics and clinical characteristics.

	Variable	*n* (%)	Patients (*n* = 104)
			Mean ± standard deviation
	Age at diagnosis (years)		45 ± 16.9

Gender	Male	73 (70.2)	
	Female	31 (29.8)	

Race	Caucasian	85 (81.7)	
	Aboriginal	10 (9.6)	
	Others	9 (8.7)	
	Smoking	27 (26.0)	
	Hypertension	63 (60.6)	
	Diabetes	15 (14.4)	
	Hypercholesterolaemia	31 (29.8)	
	Statin at presentation	26 (25.0)	
	Additional renal pathology on biopsy	23 (22.1)	
	Nephrotic syndrome	13 (12.5)	
	Haematuria	87 (83.7)	
	Pyuria	33 (31.7)	
	Baseline proteinuria (g/day)		2.5 ± 2.7
	Creatinine at diagnosis (μmol/L)		161.9 ± 139.6
	eGFR at diagnosis (mL/min)^a^		57.1 ± 27.1
	Immunosuppression used	21 (20.2)	

Risk group	A: low risk	25 (24.0)	
	B: intermediate risk	58 (55.8)	
	C: high risk	21 (20.2)	

Mesangial cellularity	M0	36 (34.6)	
	M1	68 (65.4)	

Endocapillary proliferation	E0	87 (83.7)	
	E1	17 (16.3)	

Segmental sclerosis	S0	51 (49.0)	
	S1	53 (51.0)	

Interstitial fibrosis/tubular atrophy	T0	51 (49.0)	
	T1	42 (40.4)	
	T2	11 (10.6)	

Crescents	C0	80 (76.9)	
	C1	18 (17.3)	
	C2	6 (5.8)	

^a^Estimated glomerular filtration rate as measured by the CKD-EPI equation.

**Table 2:** Univariate analysis of risk factors for progression.

Univariate analysis of risk factors for progression	Variable	Combined patients (*n* = 104)				
		Did not progress to kidney failure *n* (%)	Progressed to kidney failure *n* (%)	Unadjusted hazard ratio	95% CI	*p* value
	No. of patients	76	28			—

Gender	Male	50 (68.5)	23 (31.5)	Ref		
	Female	26 (83.9)	5 (16.1)	0.4	0.2, 1.1	0.075

Race	Caucasian	61 (71.8)	24 (28.2)	Ref		
	Aboriginal	8 (80.0)	2 (20.0)	1.5	0.3, 6.2	0.603
	Others	7 (77.8)	2 (22.2)	1.1	0.2, 7.8	0.933

Smoking	Yes	19 (70.4)	8 (29.6)	1.2	0.5, 2.9	0.654
	No	57 (74.0)	20 (26.0)	Ref		

Hypertension	Yes	58 (66.7)	29 (33.3)	1.9	0.8, 4.4	0.161
	No	34 (82.9)	7 (17.1)	Ref		

Diabetes	Yes	9 (60.0)	6 (40.0)	1.5	0.6, 3.8	0.349
	No	67 (75.3)	22 (24.7)	Ref		

Hypercholesterolaemia	Yes	24 (77.4)	7 (22.6)	0.7	0.3, 1.7	0.437
	No	52 (71.2)	21 (28.8)	Ref		

Statin at presentation	Yes	19 (73.1)	7 (26.9)	1.0	0.4, 2.8	0.963
	No	57 (73.1)	21 (26.9)			

Additional renal pathology on biopsy	Yes	13 (56.5)	10 (43.5)	2.9	1.3, 6.4	0.008⁣^∗^
	No	63 (77.8)	18 (22.2)	Ref		

Nephrotic syndrome	Yes	9 (69.2)	4 (30.8)	1.0	0.4, 3.0	0.953
	No	67 (73.6)	24 (26.4)	Ref		

Haematuria	Yes	63 (72.4)	24 (27.6)	1.2	0.4, 3.3	0.798
	No	13 (76.5)	4 (23.5)	Ref		

Pyuria	Yes	22 (66.7)	11 (33.3)	1.2	0.6, 2.7	0.626
	No	54 (76.1)	17 (23.9)	Ref		

Baseline proteinuria	< 1 g/day	35 (87.5)	5 (12.5)	Ref		
	1–3 g/day	25 (75.8)	8 (24.2)	2.2	0.7, 6.7	0.169
	> 3 g/day	14 (48.3)	15 (51.7)	4.3	1.6, 12.0	0.005⁣^∗^
	Proteinuria at baseline (g/day)^§^	1.07 (0.4, 2.8)	3.9 (1.2, 6.3)	1.2	1.1, 1.3	0.003⁣^∗^

Immunosuppression used	Yes	14 (66.7)	7 (33.3)	1.5	0.6, 3.6	0.458
	No	62 (74.7)	21 (25.3)	Ref		

Risk group	A: low risk	25 (100)	0 (0)	N.A		
	B: intermediate risk	42 (72.4)	16 (27.6)	0.3	0.1, 0.7	0.002⁣^∗^
	C: high risk	9 (42.9)	12 (57.1)	Ref		

Mesangial cellularity	M0	32 (88.9)	4 (11.1)	Ref		
	M1	44 (64.7)	24 (35.3)	3.6	1.3, 10.5	0.018⁣^∗^

Endocapillary proliferation	E0	65 (74.7)	22 (25.3)	Ref		
	E1	11 (64.7)	6 (35.3)	1.7	0.7, 4.1	0.276

Segmental sclerosis	S0	41 (80.4)	10 (19.6)	Ref		
	S1	35 (66.0)	18 (34.0)	1.6	0.7, 3.5	0.247

Interstitial fibrosis/tubular atrophy	T0	42 (82.4)	9 (17.6)	Ref		
	T1	30 (71.4)	12 (28.6)	1.5	0.61, 3.6	0.388
	T2	4 (36.4)	7 (63.6)	8.9	3.1, 25.5	0.001⁣^∗^

Crescents	C0	60 (75.0)	20 (25.0)	Ref		
	C1	11 (61.1)	7 (38.9)	1.5	0.6, 3.6	0.352
	C2	5 (83.3)	1 (16.7)	1.0	0.1, 7.2	0.967

*Note: n* (%) for categorical variables. Mesangial cellularity, M0: < 50% of glomeruli; M1: > 50% of glomeruli and endocapillary proliferation; E0: absence of hypercellularity; E1: hypercellularity in any glomeruli and segmental sclerosis; S0: absence of sclerosis; S1: segmental sclerosis in any glomeruli and interstitial fibrosis/tubular atrophy; T0: nil (0%–25%); T1: 25%–50%; T2: > 50%, crescents; C0: No crescents; C1: crescents in less than 1/4 glomeruli; C2: crescents in > 1/4 of glomeruli.

^§^Median (25th and 75th percentile) for skewed variables.

⁣^∗^*p* < 0.05.

**Table 3:** Risk factors for progression to kidney failure by multivariate (Cox regression) analysis.

	Risk factors for progression to kidney failure by multivariate (cox regression) analysis				95.0% CI	
	Regression coefficient (B)	Standard error	*p* value	Hazard ratio	Lower	Upper
Mesangial cellularity > 50% glomeruli (M1)	1.00	0.61	0.095	2.63	0.85	8.15
Interstitial fibrosis/tubular atrophy 25%–50% (T1)	−0.20	0.48	0.674	0.82	0.32	2.09
Interstitial fibrosis/tubular atrophy > 50% (T2)	1.95	0.56	0.001⁣^∗^	7.00	2.32	21.05
Additional renal pathology on biopsy	1.36	0.44	0.002⁣^∗^	3.90	1.63	9.29
Baseline proteinuria	0.14	0.06	0.024⁣^∗^	1.15	1.02	1.29

^∗^ *p* value < 0.05 = denotes statistical significance.

**Table 4:** Comparison of renal survival and risk factors for kidney failure among previous cohort studies.

Author and year of publication	Nation	Cohort size	5-year renal survival (%)	10-year renal survival (%)	Follow-up period	Variables associated with kidney failure
Present study (2023)	Australia	104	84	74	2000–2020	T2, presence of additional pathology on biopsy and increased proteinuria

Nicholls et al. (1984) [7]	Australia	244	91.5	87.5	1959–1971	Persistent microscopic and macroscopic haematuria, proteinuria, hypertension, crescents and reduced eGFR at diagnosis
Ibels et al. (1997) [8]	Australia	174	92	88		Nephrotic range proteinuria and reduced eGFR at diagnosis

Koyama et al. (1997) [14]	Japan	335	96	85	1985–1993	Increased proteinuria, reduced eGFR at diagnosis and hypertension

Geddes et al. (2003) [6]	Australia	121		87	1959–1993	Younger age and increased proteinuria
	UK	112		64	1977–1995	
	Canada	274		62	1963–1997	
	Finland	204		96	1980–1995	

Chacko et al. (2005) [9]	India	478	55	33	1994–2003	Hypertension and nephrotic range proteinuria

Komatsu et al. (2009) [11]	Japan	304		75 in early diagnosis group (1981–1995) 96 in late diagnosis group (1996–2006)	1981–2006	Increased proteinuria, reduced eGFR at diagnosis, hypertension and late presentation

Le et al. (2011) [12]	China	1155		83	1989–2005	Increased proteinuria, reduced eGFR at diagnosis, hypertension, high uric acid and low serum protein

Lee et al. (2012) [13]	Korea	1364		96	1979–2008	Increased proteinuria, reduced eGFR at diagnosis, hypertension, segmental sclerosis ≥ 20% S1 and low serum protein

Moriyama et al. (2014) [10]	Japan	1012		84	1974–2011	Increased proteinuria, reduced eGFR at diagnosis and high uric acid

Imai et al. (2020) [23]	Japan	267	93	84	1985–2004	Reduced eGFR at diagnosis, hypertension and smoking

## Data Availability

Data are available on request due to privacy/ethical restrictions.
